# Impaired Clearance and Enhanced Pulmonary Inflammatory/Fibrotic Response to Carbon Nanotubes in Myeloperoxidase-Deficient Mice

**DOI:** 10.1371/journal.pone.0030923

**Published:** 2012-03-30

**Authors:** Anna A. Shvedova, Alexandr A. Kapralov, Wei Hong Feng, Elena R. Kisin, Ashley R. Murray, Robert R. Mercer, Claudette M. St. Croix, Megan A. Lang, Simon C. Watkins, Nagarjun V. Konduru, Brett L. Allen, Jennifer Conroy, Gregg P. Kotchey, Bashir M. Mohamed, Aidan D. Meade, Yuri Volkov, Alexander Star, Bengt Fadeel, Valerian E. Kagan

**Affiliations:** 1 Center for Free Radical and Antioxidant Health, University of Pittsburgh, Pittsburgh, Pennsylvania, United States of America; 2 Department of Environmental and Occupational Health, University of Pittsburgh, Pittsburgh, Pennsylvania, United States of America; 3 Pathology and Physiology Research Branch, Health Effects Lab Division, National Institute for Occupational Safety and Health, Morgantown, West Virginia, United States of America; 4 Departments of Cell Biology and Physiology, University of Pittsburgh, Pittsburgh, Pennsylvania, United States of America; 5 Department of Clinical Medicine, Trinity College, Dublin, Ireland; 6 Center for Research on Adaptive Nanostructures and Nanodevices, Trinity College, Dublin, Ireland; 7 School of Physics, College of Science and Health, Dublin Institute of Technology, Dublin, Ireland; 8 Department of Chemistry, University of Pittsburgh, Pittsburgh, Pennsylvania, United States of America; 9 Division of Molecular Toxicology, Institute of Environmental Medicine, Karolinska Institutet, Stockholm, Sweden; National Institutes of Health, United States of America

## Abstract

Advancement of biomedical applications of carbonaceous nanomaterials is hampered by their biopersistence and pro-inflammatory action *in vivo*. Here, we used myeloperoxidase knockout B6.129X1-MPO (MPO k/o) mice and showed that oxidation and clearance of single walled carbon nanotubes (SWCNT) from the lungs of these animals after pharyngeal aspiration was markedly less effective whereas the inflammatory response was more robust than in wild-type C57Bl/6 mice. Our results provide direct evidence for the participation of MPO – one of the key-orchestrators of inflammatory response – in the *in vivo* pulmonary oxidative biodegradation of SWCNT and suggest new ways to control the biopersistence of nanomaterials through genetic or pharmacological manipulations.

## Introduction

Biopersistence of carbon nanotubes (CNT) - resulting from their inherent durability [1], [2] is one the major stumbling blocks on the way of their broad biomedical applications. This is because engineered CNT represent a possible health risk due to their ability to cause pulmonary inflammation, severe oxidative stress and early onset fibrosis in animals [3], [4], [5], [6]. They also exert genotoxic effects [7] possibly associated with carcinogenesis (e.g., induction of mesotheliomas) [8], [9]. These health concerns have been associated – to a large extent – with the reported long “life-span” of SWCNT in the lung thus necessitating exploration of possible metabolic pathways leading to their biodegradation.

Although different types of chemical oxidative cutting of CNT - lengthwise and shortening – have been described, they require harsh oxidants (e.g., sulphuric acid plus H_2_O_2_ or KMnO_4_) [10]. Recently, we reported that reactive intermediates of horseradish peroxidase are effective in enzymatic oxidative biodegradation of CNT and graphene oxide [11], [12], [13]. Moreover, myeloperoxidase (MPO), an abundant enzyme of inflammatory cells (neutrophils), - involved in the principal defense mechanisms of innate immunity -was also effective in oxidative biodegradation of CNT in biochemical models and in cells yielding the products that did not cause pulmonary inflammation in mice [14]. However, the relevance of this mechanism for CNT biodegradation *in vivo* was lacking. Here, we employed MPO knockout B6.129X1-MPO (MPO k/o) *vs* wild-type C57Bl/6 mice (w/t) and demonstrated that clearance and oxidation of single wall carbon nanotubes (SWCNT) in the lungs after their pharyngeal aspiration was markedly less effective whereas inflammatory response was more robust in the former than in the latter. Our results provide direct evidence for the participation of MPO in pulmonary biodegradation of SWCNT *in vivo*. Based on these data, new genetic and pharmacological approaches can be developed to regulate the biopersistence of nanomaterials in tissues.

## Results and Discussion

### Pulmonary inflammatory responses in w/t and MPO k/o mice exposed to SWCNT

**Figure 1:**
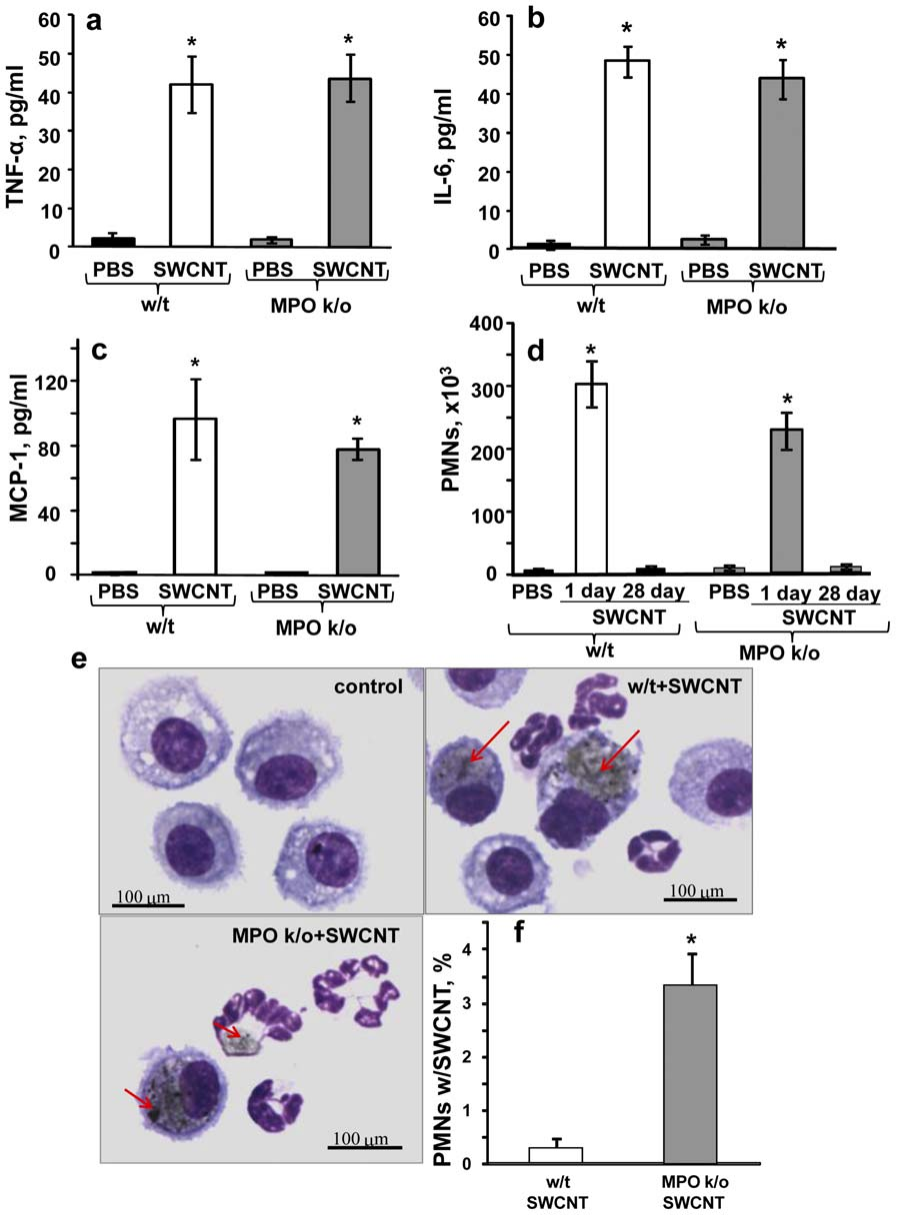
Characterization of pulmonary inflammatory responses to SWCNT in w/t and MPO k/o mice at day 1 after pharyngeal aspiration exposure. a–c. Levels of pro-inflammatory cytokines (a - TNF-α; b - IL-6; c – MCP-1) in BAL fluid of w/t and MPO k/o mice. Mean ± SEM (n = 6 mice/group). *p<0.05, *vs* control PBS-exposed mice. d. Content of PMNs in BAL fluid of w/t and MPO k/o mice. Mean ± SEM (n = 6 mice/group). *p<0.05, *vs* control PBS-exposed mice. e. Typical microscopic images of inflammatory cells in BAL fluid with SWCNT inclusions (red arrows). f. Content of PMNs with engulfed SWCNT in BAL fluid of w/t and MPO k/o mice. Mean ± SEM (n = 6 mice/group). *p<0.05, *vs* w/t mice.

Upon pharyngeal aspiration, SWCNT elicited typical inflammatory responses in w/t and MPO k/o mice documented by increased production of pro-inflammatory cytokines (TNF-α, IL-6 and MCP-1) 1 day after the exposure (Fig. 1a, b, c). BAL cytology indicated a robust and early (day 1) accumulation of neutrophils - slightly weaker in MPO k/o mice *vs* w/t animals (Fig. 1d), followed by a sequential appearance of macrophages in both groups of animals (with a peak at day 7) (data not shown) [4]. At day 28 post exposure, the amounts of PMN in BAL fluid from exposed MPO k/o mice and w/t mice are not different from those in the respective control groups of animals (Fig. 1d). In BAL, phagocytized SWCNT were detected inside PMNs and macrophages (Fig. 1e). Because of the enrichment of PMNs with MPO, we compared the SWCNT content in these cells. An ∼10 times greater number of PMNs from MPO k/o mice had SWCNT inclusions than those from w/t animals (Fig. 1f), in agreement with the lack of MPO-driven biodegradation in MPO k/o mice.

**Figure 2:**
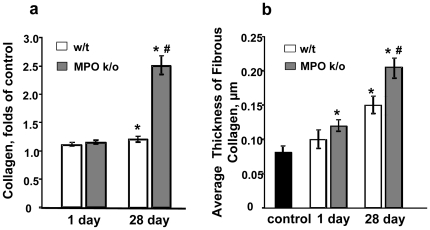
Changes in the content of collagen and average thickness of alveolar connective tissue in the lungs of w/t and MPO k/o mice at days 1 and 28 after pharyngeal aspiration of SWCNT. a. Accumulation of collagen in the lung of w/t or MPO k/o mice. Mean ± SEM (n = 6 mice/group). *p<0.05, *vs* control PBS-exposed mice, # p<0.05, *vs* w/t mice 28 days post exposure. b. Morphometric assessments of average thickness of alveolar connective tissue in the lung of w/t or MPO k/o mice. Mean ± SEM (n = 6 mice/group). *p<0.05, *vs* control PBS-exposed mice, # p<0.05, *vs* w/t 28 days post exposure.

Assessments of fibrosis by measurements of collagen deposition on day 28 post-exposure revealed its significantly higher amounts in the lungs of MPO k/o mice compared to w/t animals (Fig. 2a). A stronger fibrogenic response in k/o *vs* w/t mice was also evident from quantitative morphometry of the thickness of fibrous collagen in the alveolar connective tissue (Fig. 2b). An increase of the thickness was observed already at day 1 post-exposure and progressed further by day 28 compared with air control groups. A markedly higher collagen content of the alveolar wall was detected in the lung of MPO-deficient mice compared to w/t animals (Fig. 2b).

### Assessment of SWCNT aggregates in the lung of w/t and MPO k/o mice

**Figure 3:**
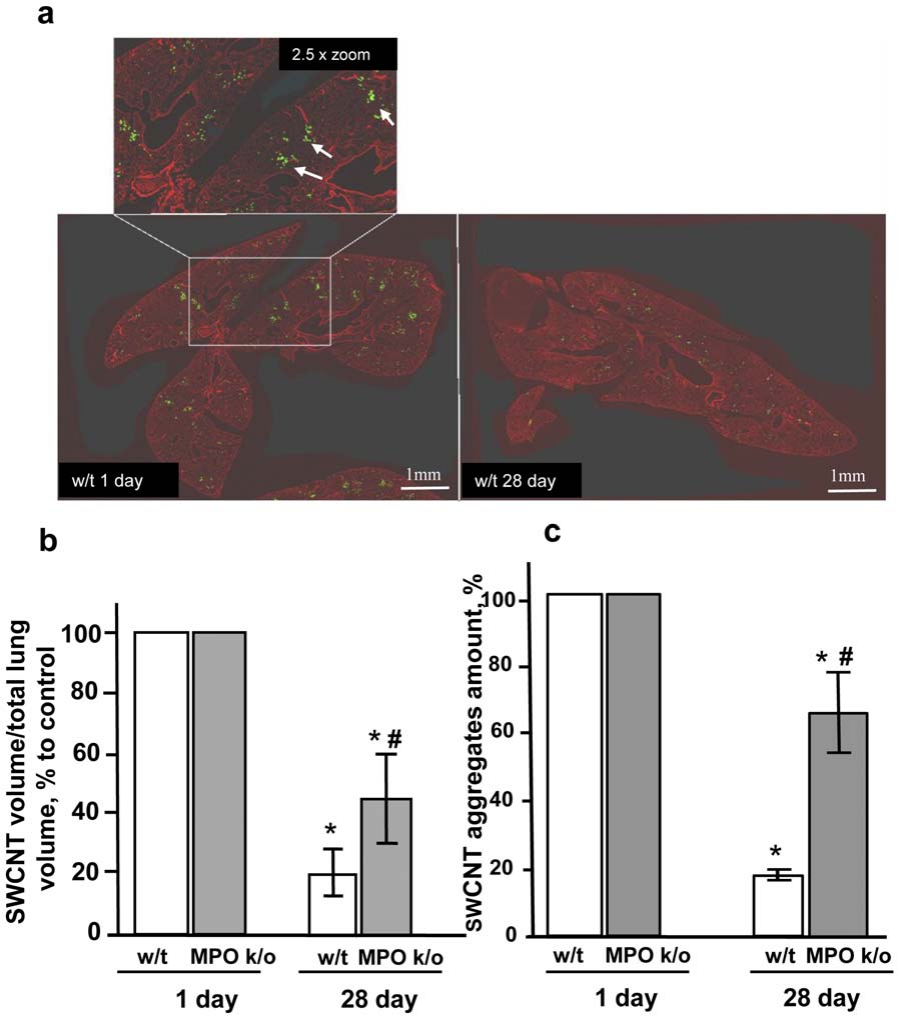
Assessment of the volume of SWCNT aggregates in the lung tissue sections from w/t and MPO k/o mice. a. Representative images of the lung tissue sections. Insert - higher magnifigation (2.5× zoom) of a field illustrating the presence of SWCNT (green punctuate spots pointed by white arrows). b. Quantitation of SWCNT aggregates (SWCNT volume/total lung volume) using their specific absorbance (750–850 nm), * p<0.05, *vs* w/t 1 day post exposure, # p<0.05, *vs* w/t 28 days post exposure. c. Assessment of SWCNT aggregates - number per microscopic field - using an automated IN Cell Analyser 1000 microscope, * p<0.05, *vs* w/t 1 day post exposure, # p<0.05, *vs* w/t 28 days post exposure.

To evaluate the content of SWCNT and their aggregates we employed quantitative imaging by illuminating lung sections with the light in the spectral range of 750–840 nm selectively absorbed by SWCNT. In the images presented in Fig. 3a, the lung tissue is shown in red, the SWCNT image was inverted such that the nanotubes look bright and pseudocolored in green. The volume occupied by SWCNT at day 1 post-exposure (∼07–1.0% of the total lung volume) markedly decreased by day 28 (to 0.02–0.03%). Elimination of SWCNT from the lung of w/t mice was almost 2 times more effective than in MPO k/o animals (Fig. 3b). Additionally, we employed another approach for collecting SWCNT images: we scanned unstained paraffin embedded lung sections using INCell Analyser 1000 high content screening; optically detectable SWCNT aggregates were counted in each field throughout the lung sections. The content of SWCNT aggregates in each field on day 1 post-exposure was approximately the same in both groups of mice but markedly decreased by day 28 after exposure (Fig. 3c). Assuming the content of aggregates on day 1 in both groups as 100%, the relative content of SWCNT aggregates dropped to <20% in w/t samples but remained at the level of >60% in the MPO k/o lung sections – a more than 3-fold difference.

**Figure 4:**
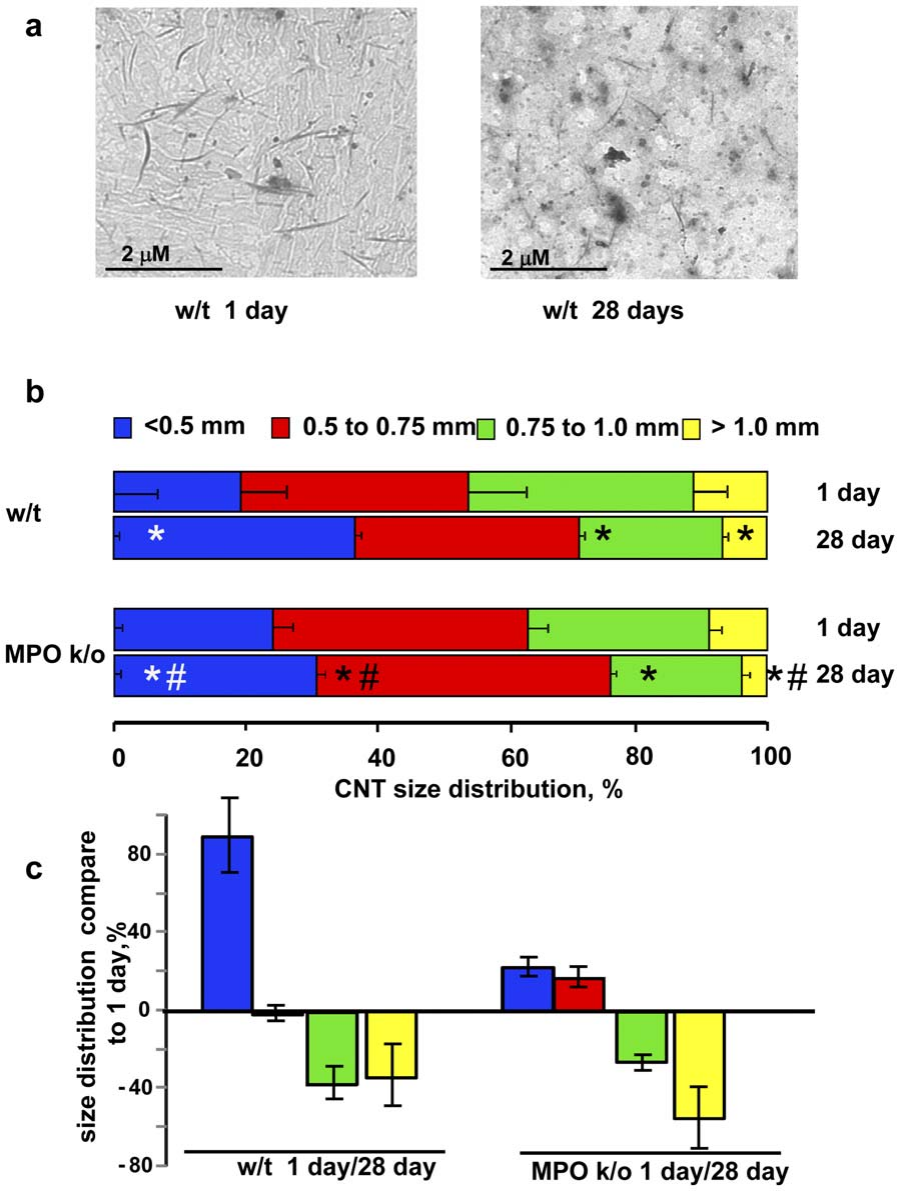
Evaluation of SWCNT size distribution in solubilized lungs of w/t and MPO k/o mice at days 1 and 28 post exposure by t ransmission electron **microscopy.** a. Typical TEM images of SWCNT after solubilization of the lung tissue. b. Size distribution of SWCNT present in the lung at days 1 and 28 post exposure. * p<0.05, *vs* w/t 1 day post exposure, # p<0.05, *vs* w/t 28 days post exposure. c. Changes in size distribution of SWCNT at day 28 post exposure compared to day 1 post exposure.

To obtain further evidence for “cutting” of SWCNT by MPO, we performed TEM analysis of the lung samples after solubilization of the tissue and evaluated the size distribution of SWCNT and their aggregates (Fig. 4). On day 1 after exposure, ∼35–40% of SWCNT were represented by large aggregates of >0.75 µm whereas 20–25% of aggregates were >0.5 µm and 35–40% had the size range from 0.5–0.75 µm in w/t and k/o animals. This distribution had changed markedly at day 28 after exposure in favour of small aggregates. The “cutting” pattern was different in w/t and k/o animals: a ∼2-fold increase for aggregates <0.5 µm, mostly at the expense of the decreased amount of large aggregates, was observed in the lungs of w/t mice whereas these changes in the SWCNT size distribution were much less pronounced in MPO k/o mice (Fig. 4b, c). This suggests that MPO was involved in the cutting of SWCNT into smaller fragments - in accord with our previous *in vitro* experiments [14].

### Raman spectroscopic evaluation of defects in SWCNT in the lungs of w/t and MPO k/o mice

**Figure 5:**
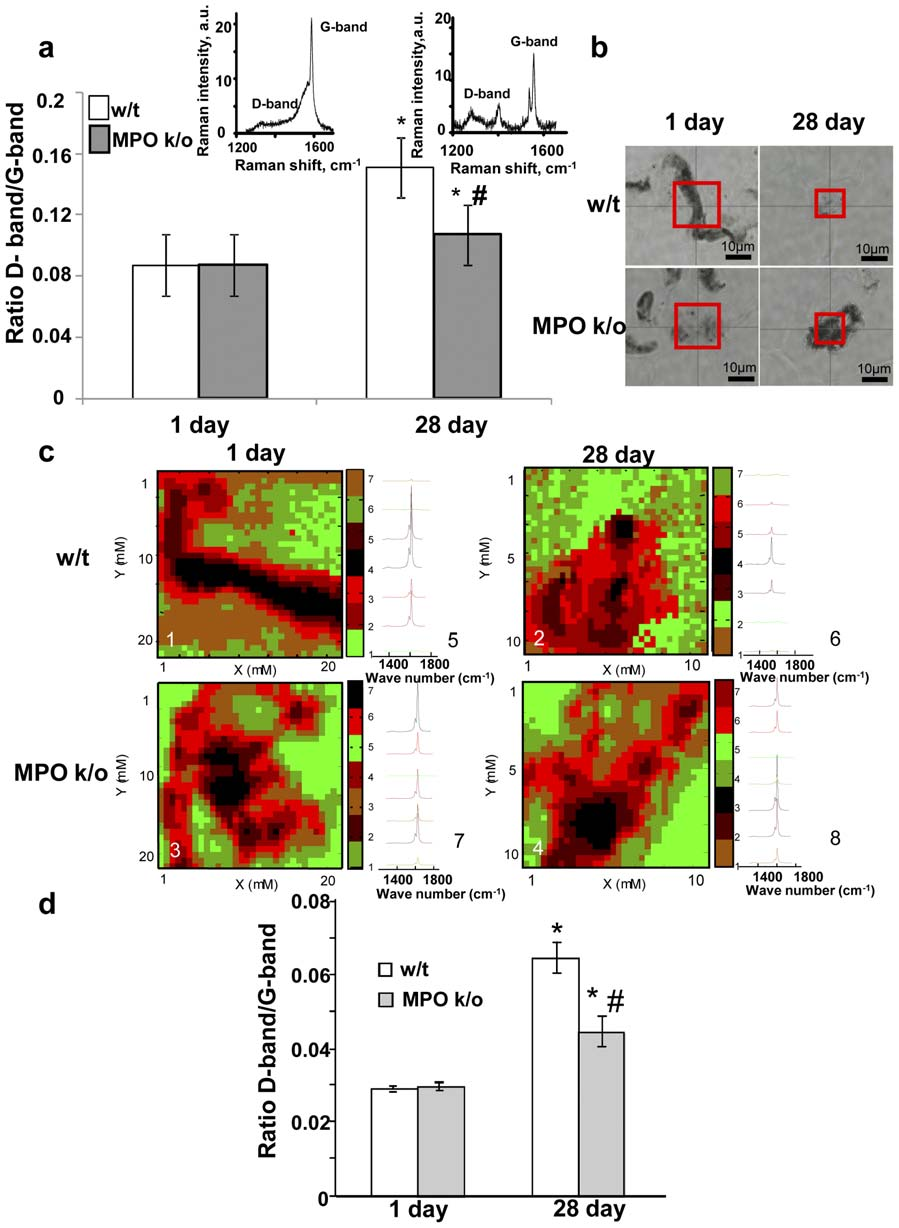
Raman spectroscopic evaluation of “oxidative” defects in SWCNT present in the lungs of w/t and MPO k/o mice at days 1 and 28 post exposure using single point Raman spectroscopy or Raman mapping of different areas within the tissue samples. a. D-band/G- band ratios for single point Raman spectra obtained from samples at days 1 and 28 post exposure. * p<0.05, *vs* w/t 1 day post exposure, # p<0.05, *vs* w/t 28 days post exposure. Insert – typical Raman spectra (excitation at 633 nm) of solubilized lungs of w/t mice at days 1 and 28 post exposure. b–d. Raman mapping of SWCNT in different areas of the lung sections. b. Examples of bright-field images with a red box indicating the area where 32×32 Raman spectra were acquired. Note that the sizes of the acquired areas were different at day 1 and day 28 (20 µm×20 µm and 10 µm×10 µm respectively), as significantly smaller SWCNT aggregates were detected at day 28 in w/t mice and the scanned areas were adjusted accordingly. c. Raman maps (excitation at 473 nm excitation) with examples of spectra corresponding to each of the clusters. d. D-band/G-band ratios for Raman spectral maps obtained from the lung of w/t and MPO k/o mice at days 1 and 28 post exposure.

MPO-driven biodegradation occurs via oxidative modification of SWCNT with the appearance of characteristic defects detectable by Raman spectroscopy [14]. Therefore, we utilized two different Raman spectroscopic techniques to determine whether these oxidation-associated defects could be detected in the samples of the lung from exposed mice. First, we performed Raman spectroscopy after solubilization of the lung tissue. To assess oxidative biodegradation of SWCNT, the D-band/G-band ratios were calculated, whereby an increase in the ratio indicates oxidative degradation [15], [16]. Typical Raman spectra documenting the signals from non-oxidized SWCNT with a pronounced G band and a very weak D-band were recorded on day 1 post-exposure resulting in low D-band/G-band ratios for both w/t and MPO k/o animals (Fig. 5a). On day 28 post-exposure, the D-band/G-band ratio remained almost unchanged for the samples from k/o animals whereas an ∼2-fold increase was found in the lungs of w/t mice. Next, we obtained Raman maps of ten different areas that contained SWCNT within each lung tissue section. Fig. 5b shows examples of bright-field images of samples from w/t and MPO k/o mice, respectively, at day 1 post-exposure, with a red box indicating the area where the Raman maps were acquired; (5c, 1–4) shows typical Raman maps obtained based on the clusters (5c, 5–8) per pixel. These bright-field images and Raman maps were generated for every sample on days 1 and 28 after the SWCNT exposure. SWCNT degradation increased over time. Importantly, 28 days post exposure there was a significant difference (p = 0.0277) between the D-band/G-band ratios of the SWCNT indicative of a markedly higher SWCNT degradation in w/t mice than in MPO k/o animals (Fig. 5d).

While the difference in clearance of SWCNT from the lungs of w/t and MPO k/o mice was significant, it is also obvious that additional, yet to be identified, factors contribute to biodegradation and elimination of SWCNT. Indeed, SWCNT clearance occurred, although at a lower rate, in MPO k/o animals. Contribution of other heme-peroxidases to the clearance of SWCNT is quite possible. Degradation of SWCNT by MPO during the peroxidase cycle whereby the reaction with H_2_O_2_ yields a reactive intermediate, compound I, which oxidizes chloride by a single 2-electron transfer to produce the hypochlorous acid (HOCl). Both oxidants – reactive MPO intermediates and hypochlorous acid – are involved in the oxidative degradation of nanotubes [14] due to the breakage of C-C and C-H bonds [17]. Reactive intermediates can be also generated by other heme-peroxidases such as eosinophil peroxidase, lactoperoxidase, thyroid peroxidase as well as by hemoproteins with peroxidase activity (hemoglobin, myoglobin, cytochromes P450). In fact, the ability of horseradish peroxidase (HRP) and lactoperoxidase to oxidatively biodegrade SWCNT *in vitro* has been documented [11], [12], [18]. While it is possible that alternative peroxidase pathways may participate in pulmonary biodegradation of SWCNT the results of the current study - demonstrating a significantly lower clearance of SWCNT from the lungs of MPO k/o mice *vs* w/t animals - are supportive of the essential role of MPO in this process. While this study utilized SWCNT, it is highly likely that multi-walled carbon nanotubes, fullerenes, graphene and other carbonaceous particles may also undergo MPO-catalyzed modifications and degradation [19].

In contrast to *in vitro* incubation systems, non-covalent coating of SWCNT with proteins and lipids *in vivo* [20], [21] occurring during their interactions with components of biofluids will inevitably affect recognition patterns and metabolic pathways of the particles. Our recent studies indicate that in the lung, SWCNT are effectively coated with the major surfactant lipids and proteins. Hence, the nature of the nanomaterial “coronation” by proteins and/or lipids may impact on the degree of recognition and biodegradation by immune-competent cells.

The major bactericidal function of MPO via oxidative attack on pathogens has been well characterized [22], [23]. The ability of MPO to oxidatively modify organic xenobiotics and drugs has been also documented [24], [25]. Here, we describe a hitherto unknown role of MPO in the body to oxidatively biodegrade carbonaceous particles, specifically SWCNT. One can presume that this is one of the essential and very ancient catalytic roles of the enzyme aimed at oxidative degradation and clearance of different types of carbonaceous materials to which animals and humanoids have been exposed from primordial times – such as particles generated by intentional (e.g., during cooking of food or technological high temperature processing of metals and other materials) or unintentional (forest fires, volcano eruptions) exposures. With the advent of nanotechnologies, health concerns prompted exploration of endogenous metabolic pathways competent in clearance of these notoriously biopersistent, inflammogenic and genotoxic, hence potentially high risk materials, including SWCNT. The current work demonstrates that MPO is involved in SWCNT degradation in vivo thus offering new opportunities for controlled regulation of SWNT's “life-span” in tissues and circulation.

## Methods

### Preparation and characterization of SWCNT

SWCNT (CNI Inc., Houston, TX) produced by the high pressure CO disproportionation process (HiPco) technique [26] employing CO in a continuous-flow gas phase as the carbon feedstock and Fe(CO)_5_ as the iron-containing catalyst precursor, and purified by acid treatment to remove metal contaminates [27] were used in the study. Chemical analysis trace metal (iron) in SWCNT was performed at the Chemical Exposure and Monitoring Branch (DART/NIOSH, Cincinnati, OH) using nitric acid dissolution and inductively coupled plasma-atomic emission spectrometry (ICP-AES). Analysis revealed that SWCNT comprised of 0.23 weight % iron. SWCNT were routinely tested for bacterial endotoxin (LPS) contamination using the endpoint chromogenic LAL method, as previously described [28]. The mean diameter and surface area of SWCNT was 1–4 nm and 1040 m^2^/g. Surface area was determined by Brunauer, Emmett, and Teller (BET) analysis, and diameter and length was measured by TEM.

The chemical cutting of SWCNT was performed as reported previously [29]. Purified SWCNT were dispersed in 3∶1 mixture of concentrated H_2_SO_4_ and 30% aqueous H_2_O_2_ and sonicated in ultrasonic bath (Branson 1510 Sonifier®, output power of 70 W at 40 KHz) for 24 hrs at 0°C. The dispersion was then heated to 70°C for 10 min for “polishing” the nanotubes. This solution was then diluted 10-fold by deionized water and filtered through PTFE membrane (100 µm pore size). The collected sample was thoroughly washed with deionized water and vacuum dried at 110°C for 30 min. Obtained short SWCNT were dispersed in 25 mM HEPES buffer (pH 7.4; containing 150 mM NaCl) by sonication. For purity assessment and characterization of SWCNT we used several standard analytical techniques. TEM was employed to determine the length distribution (Fig. S1a). Raman spectroscopy was implemented to visualize the D and G bands (Fig. S1b). Diffuse reflectance infrared Fourier Transform spectroscopy (DRIFTS) was also performed (methods S1, Fig. S1c).

### Animals

Specific pathogen-free adult (8–10 week) female C57BL/6 mice (w/t) and B6.129X1-MPO mice (MPO k/o) were supplied by Jackson Lab (Bar Harbor, ME) and weighed 20.0±1.9 g when used. C57BL/6 mice are widely used as background for the development of transgenic/knockout models as well as for safety and efficacy testing. B6.129X1 MPO-deficient mice were created by targeted disruption of the MPO gene in RW4 embryonic stem cells. The mutant allele was transferred to the C57BL/6J background using a marker assisted screening protocol. Polymorphisms between parental strain 129/SvJ and recipient strain C57BL/6J were screened at approximately 20 cM intervals to select for the mice containing the most C57BL/6J composition. Heterozygous mice (approximately 91% C57BL/6J) were intercrossed and all experiments used 2–6-month-old female knockout, heterozygote, and wild-type littermate animals. The absence of MPO in MPO k/o mice was proved by Northern blot or Western blot analysis of bone marrow. Neutrophils and monocytes from both peripheral blood and bone marrow of MPO k/o animals failed to exhibit endogenous peroxidase activity. In contrast, eosinophils, which possess eosinophil peroxidase, demonstrated peroxidase staining [30].

Animals were housed one mouse per cage receiving HEPA filtered air in AAALAC-approved NIOSH animal facilities. All animals were acclimated in the animal facility under controlled temperature and humidity for one week prior to use. Animals were supplied with water and certified chow 7913 (Harlan Tekland, Indianapolis, IN) ad libitum, in accordance with guidelines and policy set forth by the Institute of Laboratory Animals Resources, National Research Council. All animal studies were carried in compliance with the policies of the Institute of Laboratory Animal Resources (National Research Council) and the experimental protocol (#07-AS-M-010), approved by the National Institute for Occupational Safety and Health (NIOSH) Institutional Animal Care and Use Committee.

### Experimental Design

Experimental protocols for the present study included pharyngeal aspiration exposure of w/t and MPO k/o mice to 40 µg/mouse SWCNT, while the corresponding control mice were administered sterile Ca^2+^+Mg^2+^-free phosphate-buffered saline (PBS) vehicle. Particulate instillation was performed as described in Methods S1. Mice were sacrificed on days 1 and 28 following the exposure. Inflammation was evaluated by total cell counts, cell differentials, and accumulation of cytokines in the bronchoalveolar lavage (BAL) fluid. Fibrogenic responses to exposed materials were assessed by morphometric measurements and collagen deposition.

### Obtaining bronchoalveolar lavage (BAL) from mice

Mice were sacrificed with intraperitoneal injection of sodium pentobarbital (>100 mg/kg) and exsanguinated. The trachea was cannulated with a blunted 22 gauge needle, and BAL was performed using cold sterile PBS at a volume of 0.9 ml for first lavage (kept separate) and 1.0 ml for subsequent lavages. Approximately 5 ml of BAL fluid per mouse was collected in sterile centrifuge tubes. Pooled BAL cells for each individual mouse were washed in PBS by alternate centrifugation (800×*g*, 10 min, 4°C) and resuspened. BAL aliquots were frozen at −80°C until processed. Procedures used for BAL cell counting and cytokine analysis is described in Methods S1.

### Sirius red staining

The distributions of type I and type III collagen in the lung tissue were determined by morphometric evaluation of the Sirius red-stained sections [3]. To identify collagen fibers under the microscope, deparaffinized and dehydrated lung sections were stained with F3BA/picric acid for 1–2 h, washed with 0.01N HCL for 1 min, and counterstained with Mayer's hematoxylin for 2 min [31]. Type I and III collagen stained by Sirus red was visualized, and 6 randomly selected areas were scored under polarized microscopy and average thickness of Sirius red positive connective tissues in the alveolar wall was quantitatively measured. Volume and surface density was measured using standard morphometric analyses of points and intercept counting [32]. Average thickness of the Sirius red positive connective tissues of the alveolar wall was computed from two times the ratio of volume density of points to the surface density of the alveolar wall.

### Lung collagen measurements

Whole lungs from each mouse were homogenized in 0.7 ml of 0.5 M acetic acid containing pepsin (Accurate Chemical and Scientific Corporation, Westbury, NY) with 1∶10 ratio of pepsin: tissue wet weight. Total lung collagen content was determined by quantifying total soluble collagen using the Sircol Collagen Assay kit (Accurate Chemical and Scientific Corporation, Westbury, NY).

### Solubilization of lungs

Lungs were homogenized using Biospec Products Inc, OK, USA tissue homogenizer in 500 µL of deionized water. After homogenization, 1% SDS was added and samples were heated to 100°C for 10 min. This was followed by sonication using the ultrasonic benchtop cleaner (Branson 2510, output power of 70 W at 40 KHz) for 10 min. After cooling the contents to room temperature, Tris-HCl buffer (final concentration 30 mM) (pH 8.0) was added. Finally, an 18 hrs incubation of the material with 100 µg/mL of proteinase K at 50°C was undertaken to completely solubilize the lungs.

### Raman maps

Raman maps of lung tissue sections were acquired using an NTEGRA Spectra microscopic system integrated with Raman spectrometer (NT-MDT, Russia) 473 nm cobalt laser operated at 10%, 100× oil objective, 2 s exposure of static spectra centred at 2070 cm^−1^. Ten maps (32×32 points, 20 µm×20 µm or 10 µm×10 µm) were recorded for each tissue section. The Raman maps were then imported into Matlab 7 (The Mathworks, USA) for analysis, where all of the spectra were normalized, smoothed, baseline corrected and K-means clustering was carried out. Raman spectroscopy of solubilized lung tissue as well as transmission electron microscopy was performed as described in Methods S1.

### Image analysis

Lung preparation for microscopic evaluation was performed as described in Methods S1. Two color images were collected from mounted sections of lung using a Hamamatsu Orca ER cooled CCD camera (Hamamatsu, Bridgewater, NJ) on a Nikon 90i upright microscope (Melville, NY), equipped with a high speed linear encoded xy stage (Prior Scientific, Rockland, MA), and fluorescence imaging and bright field imaging capabilities. The motorized stage coupled with Elements software (Nikon NIS, Melville, NY) allows collection of large area images which consist of multiple stitched and aligned frames. Each image was a 10×10 image field at 10× magnification. To collect the image of the lung tissue the autofluorescent signal from the paraffin embedment was detected using 530 nm excitation and 560–600 nm emission. To visualize nanotubes sections were illuminated with white light and blocking filter (750–840 nm transmission) was placed between the specimen and the camera. In this conditions nanotubes selectively absorbing light in the range 750–800 nm were seen as black spots. For image processing, the area of the lung in the section profile was used to outline the lung, and the autofluorescent signal was used to threshold and generate tissue area measurements in each section. The same section profile region was overlaid on the nanotube image and a dark objects thresholded to generate an area measurement of the nanotubes within the lung. In the final images shown in Fig. 3a the lung tissue is shown in red, the tube image was inverted (such that the tubes are now bright) and pseudocolored green. The scale bar is 1 mm.

### Detection of SWCNT aggregates

Unstained paraffin embedded lung tissue sections from MPO k/o and w/t mice were scanned using the IN Cell Analyser 1000 high content screening and analysis system to distinguish between the organic tissue and the inorganic SWCNT (under bright field imaging settings). Optically detectable SWCNT aggregates were counted and their distribution and sizes were calculated using IN Cell Investigator software.

### Statistics

The results are presented as means ± S.D. values from at least three experiments, and statistical analyses were performed by one-way ANOVA. The statistical significance of differences was set at p<0.05.

### Disclaimer

The findings and conclusions in this report are those of the authors and do not necessarily represent the views of the National Institute for Occupational Safety and Health.

## Supporting Information

Figure S1 **Characterization of SWCNT employed in the study.** a. Histogram detailing the length distribution of SWCNT. The mean length was determined to be 676±329 nm employing a sample size of 100. The insert depicts a TEM micrograph (500 nm scale bar) for the SWCNT sample. b. Raman spectrum for SWCNT; the D- and G- bands are marked on the spectrum. c. The spectrum obtained utilizing diffuse reflectance infrared Fourier Transform spectroscopy (DRIFTS). The unit for the ordinate axis is Kubelka-Munk (KM).
(TIF)

Methods S1 **Supplemental methods.**
(DOC)
