# Supplementary figures and images for: Impaired Clearance and Enhanced Pulmonary Inflammatory/Fibrotic Response to Carbon Nanotubes in Myeloperoxidase-Deficient Mice
Source: PLoS One. 2012 Mar 30;7(3):e30923. doi: 10.1371/journal.pone.0030923 (PMC3316527; doi:10.1371/journal.pone.0030923)

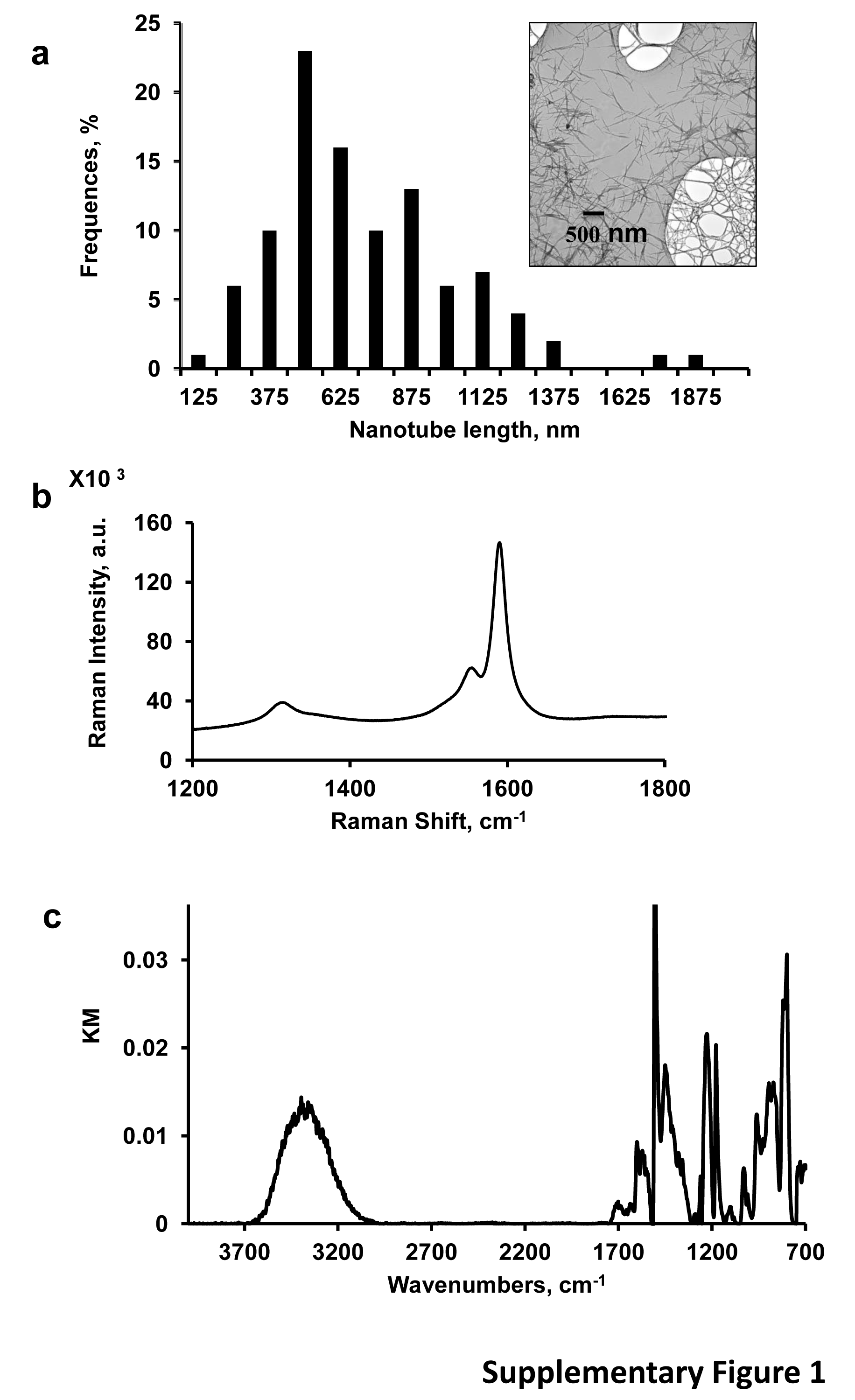

Supplement: Figure S1 — Characterization of SWCNT employed in the study. a. Histogram detailing the length distribution of SWCNT. The mean length was determined to be 676±329 nm employing a sample size of 100. The insert depicts a TEM micrograph (500 nm scale bar) for the SWCNT sample. b. Raman spectrum for SWCNT; the D- and G- bands are marked on the spectrum. c. The spectrum obtained utilizing diffuse reflectance infrared Fourier Transform spectroscopy (DRIFTS). The unit for the ordinate axis is Kubelka-Munk (KM). (TIF) [file pone.0030923.s001.tif]
