# Supplementary material for: Impaired Clearance and Enhanced Pulmonary Inflammatory/Fibrotic Response to Carbon Nanotubes in Myeloperoxidase-Deficient Mice
Source: PLoS One. 2012 Mar 30;7(3):e30923. doi: 10.1371/journal.pone.0030923 (PMC3316527; doi:10.1371/journal.pone.0030923)
Supplement: Methods S1 — Supplemental methods. (DOC) [file pone.0030923.s002.doc]

**Supporting Information**

**Impaired Clearance and Enhanced Pulmonary Inflammatory/Fibrotic Response to Carbon Nanotubes in Myeloperoxidase-Deficient Mice.**

Anna A. Shvedova^c^, Alexandr A. Kapralov^a,b^, Wei Hong Feng^a,b^, Elena R. Kisin^c^, Ashley R. Murray^c^, Robert R Mercer^c^, Claudette M. St. Croix^b^, Megan A. Lang^d^, Simon C. Watkins^d^, Nagarjun V. Konduru^a,b^, Brett L. Allen^h^, Jennifer Conroy^e^, Gregg P. Kotchey^h^, Bashir M. Mohamed^e^, Aidan D. Meade^g^, Yuri Volkov^e,f^, Alexander Star^h^, Bengt Fadeel^i^, Valerian E. Kagan^a,b*^

*From the ^a^Center for Free Radical and Antioxidant Health, University of Pittsburgh, Pittsburgh, PA, USA, ^b^Department of Environmental and Occupational Health, University of Pittsburgh, Pittsburgh, PA, USA, ^c^Pathology and Physiology Research Branch, Health Effects Lab Division, National Institute for Occupational Safety and Health, Morgantown, WV, USA, ^d^Departments of Cell Biology and Physiology University of Pittsburgh, Pittsburgh, PA, USA,  ^e^Department of Clinical Medicine, Trinity College, Dublin, Ireland,  ^f^Center for Research on Adaptive Nanostructures and Nanodevices, Trinity College Dublin, Ireland, ^g^School of Physics, College of Science and Health, Dublin Institute of Technology, Dublin, Ireland, ^h^Department of Chemistry, University of Pittsburgh, Pittsburgh, PA, USA; ^i^Division of Molecular Toxicology, Institute of Environmental Medicine, Karolinska Institutet, Stockholm, Sweden.*

*Correspondence to: Valerian E. Kagan, Ph.D., D.Sc., Center for Free Radical and Antioxidant Health, Department of Environmental and Occupational Health, University of Pittsburgh, Bridgeside Point 100 Technology Drive, Suite 350, Pittsburgh, PA, USA. Tel: 412-624-9479, Fax: 412-624-9361, E-mail: [kagan@pitt.edu](mailto:kagan@pitt.edu)

**Supplemental Methods:**

*Particulate instillation.* Mouse pharyngeal aspiration was used for particulate administration [1]. Briefly, after anesthetization with a mixture of ketamine and xylazine (62.5 and 2.5 mg/kg subcutaneous in the abdominal area), a suspension (approximately 50 µl) of SWCNT prepared in PBS (40 µg/mouse) was placed posterior on the throat and the tongue which was held until the suspension was aspirated into the lungs. Control mice were administered sterile Ca^2+^ + Mg^2+^-free PBS vehicle. The mice revived unassisted after approximately 30–40 min. All mice in SWCNT and PBS groups survived this exposure procedure. This technique provided good distribution of particles widely disseminated in a peri-bronchiolar pattern within the alveolar region as was detected by histopathology. Animals treated with the particulates or PBS recovered easily after anesthesia with no behavioral or negative health outcomes. Mice were sacrificed on days 1 and 28 following the exposures.

*BAL cell counting and differentials.* The degree of inflammatory response was estimated by quantitating total cells, macrophages, and polymorphonuclear leukocytes (PMNs) recovered by BAL. Cell counts were performed using an electronic cell counter equipped with a cell sizing attachment (Coulter model Multisizer II with a 256C channelizer, Coulter Electronics, Hialeah, FL). Alveolar macrophages and PMNs were identified by their characteristic cell shape in cytospin preparations stained with Diffquick (Fisher Scientific, Pittsburgh, PA), and differential counts of BAL cells was carried out. Three hundred cells per slide were counted.

*Lung lavage fluid cytokine analysis.* Levels of cytokines were assayed in the acellular BAL fluid following SWCNT aspiration exposures. The concentrations of TNF-α, MCP-1and IL-6 (sensitivity of assay is 5-7.3 pg/ml) were determined using the BD^TM^ Cytometric Bead Array, Mouse Inflammation kit (BD Biosciences, San Diego, CA).

*Lung preparation for microscopic evaluation.* Preservation of the lung was achieved by vascular perfusion of a glutaraldehyde (2%), formaldehyde (1%), and tannic acid (1%) fixative with sucrose as an osmotic agent [2]. This method of fixation was chosen to prevent possible disturbances of the airspace distribution of deposited materials while maintaining physiological inflation levels comparable to that of the end expiratory volume. This was performed using protocols previously employed to study pulmonary effects of SWCNT [3]. Briefly, animals were deeply anesthetized with an overdose of sodium pentobarbital, the trachea was cannulated, and laparotomy was performed. Mice were then sacrificed by exsanguination. The pulmonary artery was cannulated via the ventricle and an outflow cannula was inserted into the left atrium. In quick succession, the tracheal cannula was connected to a 5 cm H_2_O pressure source, and clearing solution (saline with 100 U/ml heparin, 350 mosM sucrose) was perfused to clear blood from the lungs. The perfusate was then switched to the fixative. Fixed lung volume was measured by water displacement [4]. Coronal sections were cut from the lungs. The lungs were embedded in paraffin and sectioned with an HM 320 rotary microtome (Carl Zeiss, Thornwood, NY).

*Fourier Transform spectroscopy (DRIFTS)* was performed employing an IR-Prestige spectrophotometer (Shimadzu Scientific, Kyoto, Japan) outfitted with an EasiDiff accessory (Pike Technologies). SWCNTs were homogeneously mixed with KBr. Using KBr as the background and taking 32 scans per sample, a spectrum was obtained over the range of 700 to 4000 cm^-1^ with a resolution of 4 cm^-1^.

*Raman spectroscopy*. Samples were prepared by drop-casting approximately 30 μL of solubilized lung material on a microscope slide and allowed to dry. A Renishaw inVia Raman microscope spectrometer (Renishaw, Gloucestershire, UK) with an excitation wavelength of 633 nm was used for all samples, spectrum was obtained over the range of 1000 to 1800 cm^-1^ to visualize D and G band intensity changes throughout the degradation process. Spectra were collected with a 15 second exposure time, at 50% laser power and averaged across 3 scans per sample.

*Transmission electron microscopy.* A FEI-Morgani TEM instrument (Tokyo, Japan) was operated at 80 KV equipped with a soft imaging system charge-coupled device (CCD) camera. TEM samples were prepared by placing of one drop of sample on a lacey carbon grid (Pacific-Grid Tech, CA,USA) and allowed to dry in ambient conditions for 2 hrs prior to TEM imaging (FEI Morgagni, 80 keV).

**References**

1. Rao GV, Tinkle S, Weissman DN, Antonini JM, Kashon ML, et al. (2003) Efficacy of a technique for exposing the mouse lung to particles aspirated from the pharynx. J Toxicol Environ Health 66: 1441-1452.

2. Mercer RR, Russell ML, Crapo JD (1994) Alveolar septal structure in different species. J Appl Physiol 77: 1060-1066.

3. Shvedova AA, Kisin ER, Mercer R, Murray AR, Johnson VJ et al. (2005) Unusual inflammatory and fibrogenic pulmonary responses to single-walled carbon nanotubes in mice. Am J Physiol Lung Cell Mol Physiol 289: L698-708.

4. Small, JV (1968) Measurements of section thickness. Proceedings of the 4th European Congress on Electron Microscopy, D.S. Bocciarelli, Editor: 609-610.
